# MaxEnt-Based Distribution Modeling of the Invasive Species *Phragmites australis* Under Climate Change Conditions in Iraq

**DOI:** 10.3390/plants14050768

**Published:** 2025-03-02

**Authors:** Nabaz R. Khwarahm

**Affiliations:** Department of Biology, College of Education, University of Sulaimani, Sulaimani 334, Kurdistan Region, Iraq; nabaz.khwarahm@univsul.edu.iq

**Keywords:** alien species, climate change, Iraq, *Phragmites australis*, machine learning spatial distribution

## Abstract

*Phragmites australis* (common reed), a recently introduced invasive species in Iraq, has swiftly established itself as a vigorous perennial plant, significantly impacting the biodiversity and ecosystem functions of Iraqi ecoregions with alarming consequences. There is an insufficient understanding of both the current distribution and possible future trends under climate change scenarios. Consequently, this study seeks to model the current and future potential distribution of this invasive species in Iraq using machine learning techniques (i.e., MaxEnt) alongside geospatial tools integrated within a GIS framework. Land-cover features, such as herbaceous zones, wetlands, annual precipitation, and elevation, emerged as optimal conditioning factors for supporting the species’ invasiveness and habitat through vegetation cover and moisture retention. These factors collectively contributed by nearly 85% to the distribution of *P. australis* in Iraq. In addition, the results indicate a net decline in high-suitability habitats for *P. australis* under both the SSP126 (moderate mitigation; 5.33% habitat loss) and SSP585 (high emissions; 6.74% habitat loss) scenarios, with losses concentrated in southern and northern Iraq. The model demonstrated robust reliability, achieving an AUC score of 0.9 ± 0.012, which reflects high predictive accuracy. The study area covers approximately 430,632.17 km^2^, of which 64,065.66 km^2^ (14.87% of the total region) was classified as the optimal habitat for *P. australis*. While climate projections indicate an overall decline (i.e., SSP126 (5.33% loss) and SSP585 (6.74% loss)) in suitable habitats for *P. australis* across Iraq, certain localized regions may experience increased habitat suitability, reflecting potential gains (i.e., SSP126 (3.58% gain) and SSP585 (1.82% gain)) in specific areas. Policymakers should focus on regions with emerging suitability risks for proactive monitoring and management. Additionally, areas already infested by the species require enhanced surveillance and containment measures to mitigate ecological and socioeconomic impacts.

## 1. Introduction

Invasive species are organisms that are introduced, either intentionally or accidentally, into ecosystems outside their natural range through environmental factors or human activities [1]. These species can adapt, spread aggressively, and dominate new habitats, leading to the displacement or extinction of native flora and fauna and causing significant ecological imbalances [2,3]. To address the threats posed by non-native species, analyzing their spatial spread is critical. The expansion of these organisms is shaped by interspecies dynamics, environmental adaptability, and variables like climate patterns, soil composition, water systems, and landscape features, with temperature and precipitation playing a pivotal role [4]. The Sixth Assessment Report of the Intergovernmental Panel on Climate Change (IPCC) highlights that average planetary temperatures have risen by nearly 1.2 °C over the last century due to human-driven warming, with projections indicating continued increases [5]. Furthermore, local and regional studies have reported that certain areas may experience varying degrees of temperature increases in the coming decades [6,7,8,9]. As such, evidence indicates that global climate change may enhance the resilience of invasive populations, enabling them to colonize broader territories and establish more dominant communities [10,11,12]. Investigating how shifting climatic conditions influence the future ranges of invasive plant species provides vital insights for designing science-backed strategies to protect ecosystems and biodiversity. Iraq has experienced significant environmental shifts linked to shifting climatic conditions, marked by frequent water shortages [13], destructive wildfires [14], land-use and land-cover change [15,16], reduced native species populations [17,18], and the increased emergence of illnesses [19]. Understanding how shifting climate patterns alter invasive species movement and developing strategies to safeguard woodland areas from their impacts is vital. Therefore, examining the causal relationship between environmental drivers and alien invasive plant species is key to evaluating broader ecosystem stability and resilience.

*Phragmites australis* is a persistent water-based grass found globally, often forming dense, single-species clusters in riverbanks and marsh ecosystems [20,21]. Recognized as an aggressive colonizer, it disrupts natural habitats in coastal zones and marshlands, though it adapts to semi-arid conditions [21]. *P. australis* aggressively outcompetes native plants for resources like moisture and soil nutrients. Thriving in shallow waters and deeper aquatic zones, it encroaches on shorelines and submerged areas. In regions like Iraq, environmental shifts and water scarcity have amplified its dominance in protected wetlands and semi-wet areas, where it exhibits remarkable salt resistance and adaptability to fluctuating hydration levels [22,23]. Its proliferation in marsh systems is altering native plant and animal habitats, threatening the ecological balance. The key ecological impacts of *P. australis* in Iraq and globally include its tendency to form monocultures in disturbed or reflooded marshes [24]. These dense stands often reduce plant diversity by outcompeting native vegetation [3,25]. Conversely, the species’ extensive root system stabilizes soil and mitigates erosion [22,23]. However, its high evapotranspiration rates may lower local water tables, though this effect remains poorly documented in Iraq. Socioeconomically, while local communities traditionally use *P. australis* for constructing homes, boats, and handicrafts, its overgrowth can impede fishing, navigation, and agricultural practices by blocking irrigation channels.

Previous studies in Iraq on *P. australis* have focused on measuring heavy metal uptake by the species [26,27] and identifying the optimal conditions for biogas production [28]. Studies on the spatial distribution of *P. australis* in Iraq are non-existent or scant; consequently, this study aims to fill this research gap. Its findings provide essential foundational data for the further investigation of alien invasive species. In this context, management actions and interventions targeting the most vulnerable landscapes could be implemented more effectively. Currently, Iraq’s approach to addressing non-native and invasive species lacks sufficient priority, and is further complicated by a limited understanding of their geographical distribution. Urgent and comprehensive research is essential to evaluate the spread of *P. australis* and the challenges posed by its proliferation to native biodiversity and ecosystem stability.

Species distribution models (SDMs), are extensively employed to evaluate the effects of environmental conditions on alien invasive species. For instance, these models utilize climatic, topographic, and edaphic drivers to estimate ecological niches under future environmental scenarios. Among these models, the maximum entropy algorithm (MaxEnt) has emerged as a prominent machine learning algorithm for simulating species–environment relationships due to its capacity to handle incomplete data and produce robust predictions [29,30]. A central question in ecological research revolves around identifying the drivers that shape species presence across specific regions. Predictive models, e.g., MaxEnt, enable the exploration of relationships between target species and their ecological requirements. They also help identify key environmental conditions that influence species occurrence in defined areas. As such, this manuscript focuses on three primary objectives: (i) mapping the current distribution of *P. australis* in Iraq and predicting its potential habitats, (ii) modeling its future distribution patterns under global climate change scenarios using MRI-ESM2.0 (SSP126 and SSP585), and (iii) identifying the key ecological factors that most significantly influence its spatial distribution.

## 2. Results

### 2.1. Model Performance

**Figure 1:**
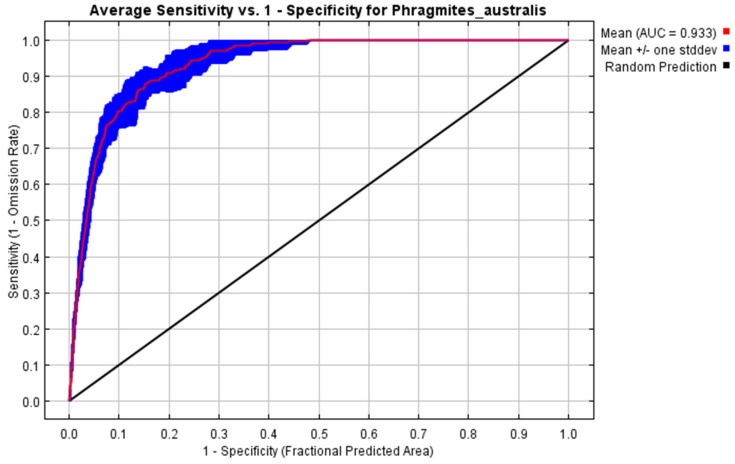
The model’s classification accuracy during validation was assessed using a metric derived from repeated training iterations. This measure evaluates the system’s capacity to correctly identify true negatives within a projected area. Across 10 computational trials, the algorithm achieved a mean value of AUC = 0.933 ± 0.012.

The assessment of habitat suitability for *P. australis* using the AUC–ROC metric demonstrated a strong performance in distinguishing favorable and unfavorable environments. Across repeated model iterations, the mean AUC value reached 0.9, with a narrow deviation of ±0.012, reflecting consistent reliability in the analysis. These outcomes, derived from 10 computational trials, highlight the model’s precision in evaluating ecological niches for *P. australis* in Iraq (Figure 1).

### 2.2. Suitability Distribution of P. australis and Conditioning Factors

**Figure 2:**
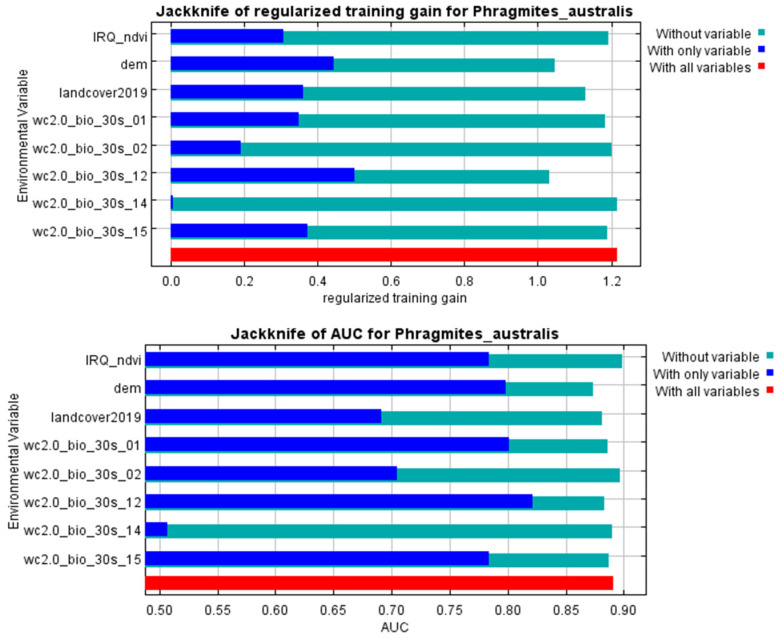
The model’s predictive improvements from constrained optimization (**top**) and classification accuracy metrics (**bottom**) were evaluated using iterative variable exclusion methods to assess how individual factors influenced habitat suitability patterns for *P. australis* in Iraq.

The ecological presence of *P. australis* in Iraq is shaped by a combination of environmental variables. Elevation and bio12 (annual precipitation) emerged as the predominant drivers, responsible for 33.2% and 31.7% of habitat distribution of *P. australis*. Secondary influences included land-cover and land-use, bio15 (precipitation seasonality), and bio1 (annual mean temperature). In addition, bio2 (mean diurnal range) and NDVI further contributed to distribution patterns, as outlined in Table 1. Cross-validation analyses using iterative testing methods reinforced these findings (Figure 2), with elevation and annual precipitation showing the strongest predictive power. In contrast, NDVI and bio14 (precipitation of driest month) exhibited a negligible influence on the habitat distribution of *P. australis*.

### 2.3. Shifts in Habitat Distribution of P. australis over Time in Iraq

**Figure 3:**
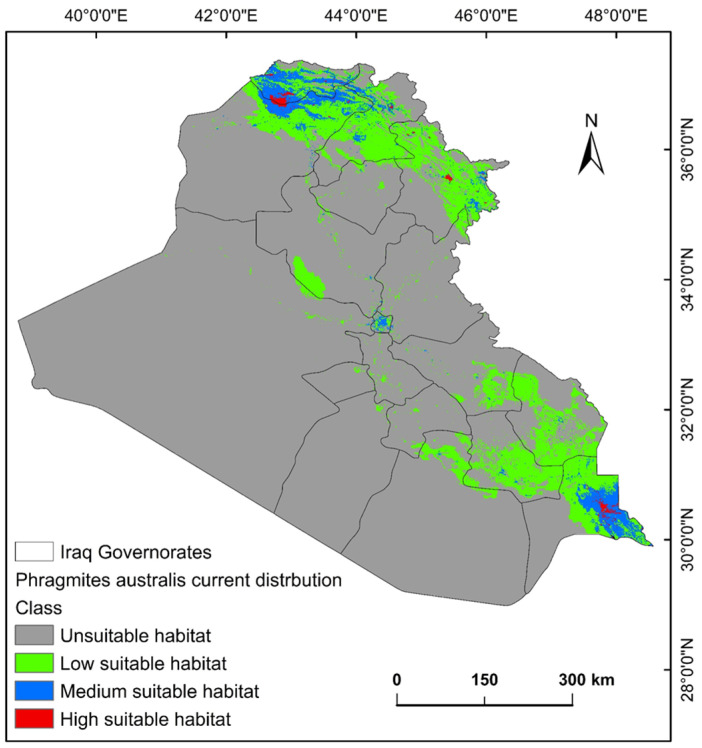
Predictive map for *P. australis* in Iraq under current climate conditions.

**Figure 4:**
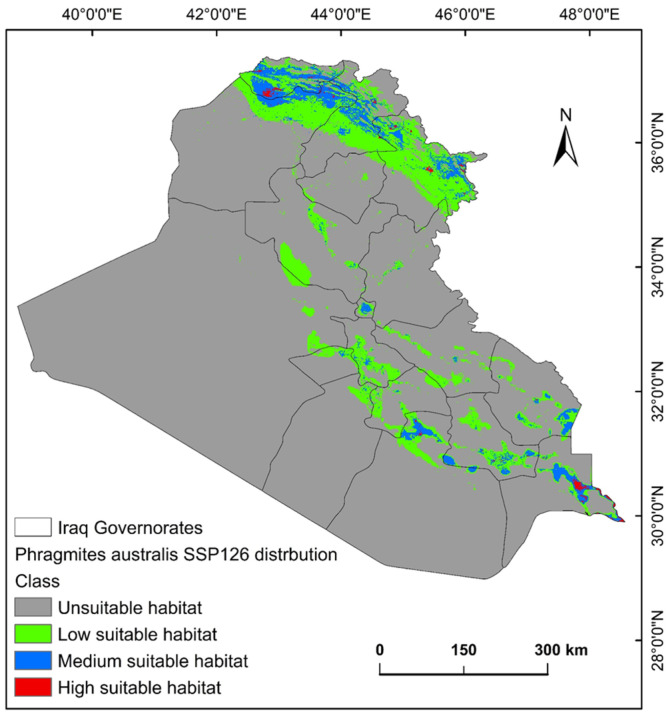
Predictive map for *P. australis* in Iraq under MRI-ESM2.0 SSP126 climate conditions.

**Figure 5:**
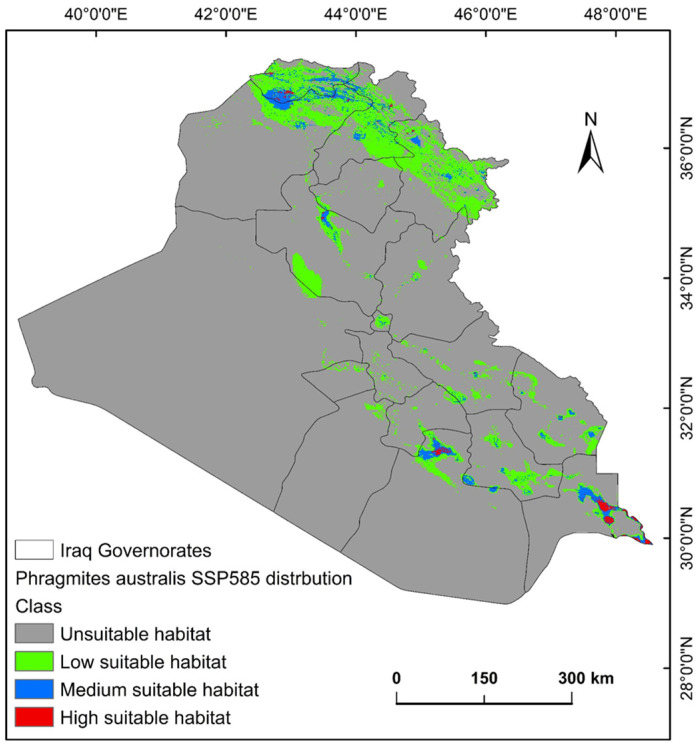
Predictive map for *P. australis* in Iraq under MRI-ESM2.0 SSP585 climate conditions.

**Figure 6:**
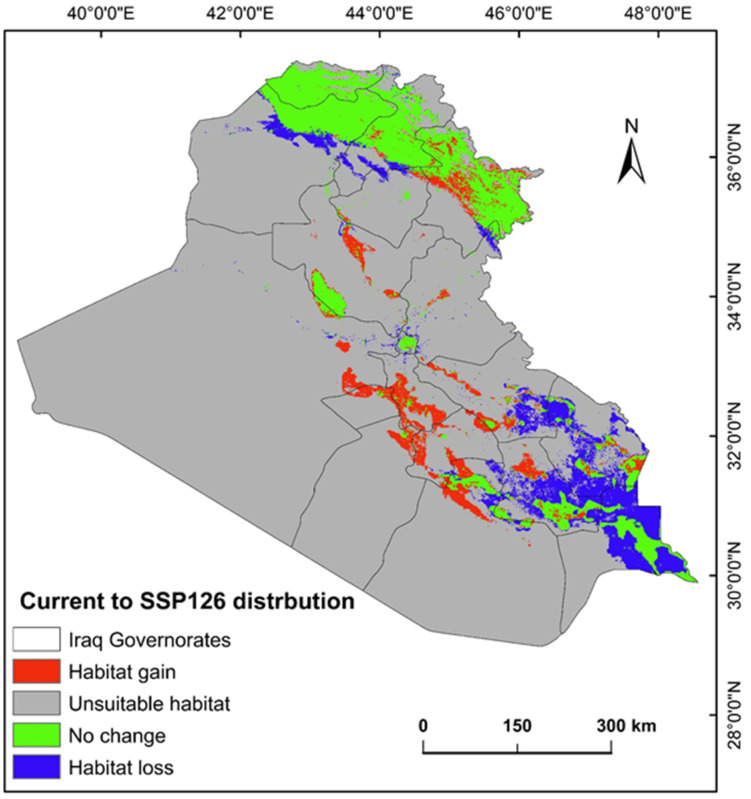
Predictive map for *P. australis* in Iraq analyzed from current prediction and future SSP126 climate scenarios.

**Figure 7:**
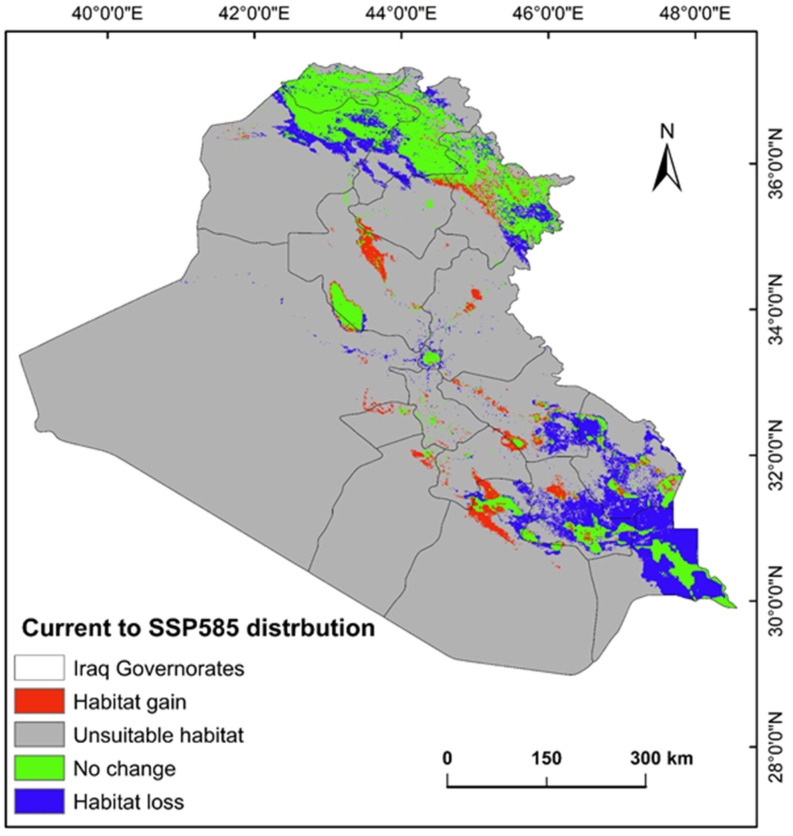
Habitat gain and loss predictive map for *P. australis* in Iraq analyzed from current prediction and future SSP585 climate scenarios.

The ecological conditions supporting *P. australis* in Iraq cover roughly 64,065.66 km^2^ (14.87%) of the total 43,0632.17 km^2^ of the national territory of Iraq. Within suitable zones, the majority (11.96% or 51,504.06 km^2^) were classified as low-probability regions, while moderate suitability accounted for 2.68% (11,540.41 km^2^), and high suitable areas constituted less than 1% (0.23% or 1021.17 km^2^). The risk zones for *P. australis* thriving are concentrated in southern Iraq, particularly across the Basra province, as well as localized sections of the Duhok, Erbil, and Sulaimani provinces in the north. Areas with moderate risk span parts of Duhok, Sulaimani, Erbil, Baghdad, and along scattered pockets of the Tigris and Euphrates waterways (Figure 3, Figure 4 and Figure 5). Conversely, extensive northern, northeastern, and western territories, including Anbar province, were deemed inhospitable for sustaining *P. australis* (Table 2). Future trends under the SSP126_2041–2060 (moderate mitigation) and SSP585_2041–2060 (high emissions) climate scenarios indicate a projected decline in suitable habitat zones for *P. australis* across Iraq. Specifically, high-suitability habitats are expected to diminish from their current coverage of 0.24% (1021.18 km^2^) to 0.23% (1008.66 km^2^) under SSP126 and 0.21% (923.09 km^2^) under SSP585 by mid-century. This contraction reflects a broader pattern of habitat degradation under intensified climatic stressors. Medium- and low-suitability zones follow analogous trajectories, with reductions in both spatial extent and ecological viability, as detailed in Table 2 and Table 3. Spatially, these shifts manifest as habitat losses and habitat gains, redistributing the species’ range across Iraq. For instance, *P. australis* is projected to recede from historically suitable areas in the south and north, while dispersing to newly viable regions in the central and northeastern parts of the country. Under the SSP126 scenario, habitat gain is estimated at 3.58% (15,403.22 km^2^), compared to a more modest 1.82% (7823.69 km^2^) under SSP585. Conversely, habitat loss is far more pronounced, reaching 5.33% (22,934.75 km^2^), under SSP126 and escalating to 6.74% (29,013.12 km^2^) under SSP585. These disparities highlight the disproportionate impact of higher emissions scenarios on habitat erosion. Visualizations of these spatial patterns, including maps of habitat loss, habitat gain, are provided in Table 3 and Figure 6 and Figure 7.

## 3. Discussion

### 3.1. Distribution of P. australis in Iraq

In Iraq, roughly 64,065.66 km^2^ (14.87%) of the whole territory offers viable conditions for *P. australis*. Within this habitable zone, high-risk areas or high suitable areas cover only 3994 km^2^ (0.9%) of the country, which are mostly located in the upper south and north (Figure 3) regions. The spatio-temporal variations are most likely due to the interesting adaptive capacity of the species to different elevations across the country. Wet regions appear conducive to the *P. australis* lifecycle. Identifying regions where the invasive species thrives is critical for forecasting outbreaks of invasion. *P. australis* survival and behavior is closely tied to localized environmental conditions, such as temperature, alongside annual precipitation (i.e., moisture levels), and land-cover in a given area. The significant contribution (nearly 85%) of precipitation, land-cover, and elevation to the distribution of *P. australis* in Iraq is demonstrated by this study. Herbaceous zones and wetlands emerged as optimal habitats, supporting the species’ invasiveness through vegetation cover and moisture retention. These findings, partly underscore the vulnerability of *P. australis* to climate change while highlighting its resilience through habitat redistribution. The interplay of CO₂ fertilization, temperature stress, and hydrological dynamics will shape its ecological impact in Iraq. By synthesizing insights from global case studies, this study advocates for a nuanced approach that balances *Phragmites* invasive threats with its ecosystem benefits, leveraging adaptive strategies to safeguard both biodiversity and wetland functionality [31,32]. These findings concur with other studies in which standard bioclimatic variables and topographic features have been used [33,34]. In addition, land-use changes caused by human activities, such as coastal development and the creation of artificial shorelines, facilitate the spread of *P. australis*. These alterations may enable different genetic variants of the reed to mix and thrive, accelerating its expansion into new areas. Simultaneously, climate change and human-induced land modifications interact to create the ideal conditions for *P. australis* growth. This, in turn, makes it more challenging to protect and restore natural wetlands, which support diverse plant and animal life [35].

### 3.2. Habitat Loss and Gain and Climate-Driven Redistribution

The results indicate a net decline in high-suitability habitats for *P. australis* under both the SSP126 (moderate mitigation; 5.33% loss) and SSP585 (high emissions; 6.74% loss) scenarios, with losses concentrated in southern and northern Iraq. This aligns with global observations of invasive *Phragmites* retreating from regions experiencing extreme aridity or salinization, as seen in North American coastal wetlands under rising temperatures [31,36]. For instance, studies [24,37] on Great Lakes wetlands found that *Phragmites* dominance declines in areas with reduced freshwater inflows, mirroring this study’s projected habitat losses in southern Iraq, where water scarcity and salinity are intensifying [24]. Conversely, the dispersal of *P. australis* to central and northeastern Iraq likely reflects adaptive colonization in regions with moderated climatic stressors, such as higher soil moisture, a pattern observed in Mediterranean and Yangtze River estuaries under similar scenarios [37]. In contrast, the disparity in habitat gains between SSP126 (3.58%) and SSP585 (1.82%) underscores the dual role of CO_2_ and temperature. Elevated CO_2_ can enhance photosynthetic efficiency and mitigate salinity stress in *Phragmites*, as demonstrated in controlled phytotron experiments [31]. However, under SSP585, extreme temperature increases may counteract these benefits, accelerating evapotranspiration and soil desiccation in newly colonized areas. This aligns with findings from Chesapeake Bay wetlands, where rising temperatures suppressed CO_2_-driven growth gains, leading to fragmented habitat viability. This study’s findings suggest that moderate mitigation (SSP126) better supports *Phragmites* expansion, likely due to reduced temperature stress on the species.

### 3.3. Implications for Biodiversity and Limitations

The projected habitat losses in southern Iraq could destabilize wetland ecosystems where *P. australis* plays a dual role as an invasive species and ecological engineer. While its monocultures often reduce native plant diversity [24], *Phragmites* also stabilizes sediments, sequesters carbon, and buffers against erosion—services critical for Iraq’s floodplain resilience [38]. The net loss of 5.33–6.74% of habitat area under both scenarios raises concerns about trade-offs: regions losing *Phragmites* may face heightened erosion, while newly colonized zones could experience biodiversity declines. Similar conflicts have been documented in the Blackwater National Wildlife Refuge, where eradication efforts improved native plant recovery but compromised shoreline stability [38]. As such, adaptive management strategies are challenging, particularly in the Tigris–Euphrates basin.

Predictive ecological maps generated by MaxEnt models come with built-in limitations including, but not limited to, input climatic drivers (both current and future global climate models) and species records quality. Moreover, temporal disparities in the input data, such as LULC, NDVI, and climate variables, may introduce bias to the results (e.g., underestimating climate-driven shifts in habitat suitability). These gaps are usually minimized by checking model boundaries, thinning species data, and carefully parameterizing the algorithm used for modeling. Uncertainties may still exist despite these fixes, especially when data are scarce. The MaxEnt model is known to work well with minimal data, but its predictions still depend on how well species records match the areas being modeled. In this study, we tackled these issues by rigorously testing geographic accuracy and using representative data.

## 4. Materials and Methods

### 4.1. Study Area

**Figure 8:**
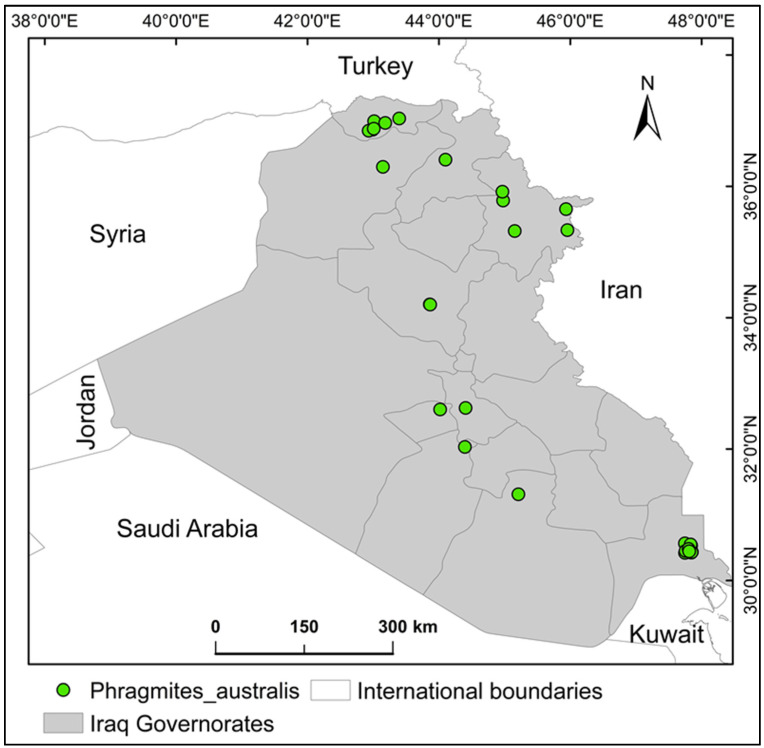
The study area (Iraq) and the occurrence records of the alien invasive species, *Phragmites australis*.

Iraq (Figure 8), encompassing approximately 430,632.17 km^2^ in the Middle East, lies between latitudes 29° N and 38° N and longitudes 39° E and 49° E. Bordered by Turkey, Iran, Kuwait, Saudi Arabia, Jordan, and Syria, the country is geographically divided into four zones: mountainous northeastern highlands, southern marsh ecosystems, western arid plains, and central transitional plateaus bridging desert and upland areas [39]. Climatic conditions vary regionally: hyper-arid in western and southern zones, semi-arid to continental in central areas, and Mediterranean in northern mountainous regions. Winter averages 16 °C, while summer extremes exceed 43 °C during daytime, cooling to 26 °C at night. Precipitation occurs mainly from December to February, though northern highlands experience rainfall from November to April [39].

### 4.2. Phragmites Australis Occurrence Records

The geographic coordinates for *P. australis* were sourced from the Global Biodiversity Information Facility (GBIF) (https://doi.org/10.15468/dl.aug5xp). These location records underwent rigorous quality evaluations using GIS tools to verify positional precision. Initial data extraction yielded 45 entries, but after applying spatial filtering techniques [40] to eliminate duplicates and ensure adequate spacing between locations (i.e., minimizing sampling bias), only 30 distinct records were retained for modeling (i.e., MaxEnt modeling). It was ensured that these records cover the spatial extent of the entire country, and that the minimum distance between two individual records was not less than 1 km [41].

### 4.3. Conditioning Factors

The model was developed using multiple environmental variables, including the standard bioclimatic variables, topographic features (e.g., elevation, vegetation proxies (e.g., NDVI)), and land-use and land-cover (LULC). These conditioning factors were chosen based on the existing literature [42,43] and insights from local experts on invasive plant species in Iraq. Climatic data, comprising 19 bioclimatic indicators for current and future scenarios, were sourced from the WorldClim database (www.worldclim.org, accessed on 1 February 2024), which provides datasets published in the Intergovernmental Panel on Climate Change (IPCC) Assessment Report 5 (AR5). These variables were originally derived from historical monthly temperature and rainfall data recorded between 1950 and 2000 at weather stations. These datasets serve as the standard baseline for climate modeling, especially in studies projecting future scenarios. The future bioclimatic variables are sourced from the global circulation model, MRI-ESM2.0 (version 2.0), from the Meteorological Research Institute of Japan. This dataset includes two scenarios, SSP 126 and SSP 585 [44]. All data have a spatial resolution of 30 arc-seconds (~1 km) and are available globally [45]. Elevation data were acquired from the Shuttle Radar Topography Mission (SRTM; http://srtm.csi.cgiar.org/srtmdata, accessed on 27 December 2024). LULC data (100-m resolution, 2019) were sourced from Copernicus Land Cover (https://zenodo.org/communities/copernicus-land-cover, accessed on 13 December 2024). Additionally, vegetation dynamics were assessed using the Normalized Difference Vegetation Index (NDVI), calculated from Landsat 8 satellite imagery (https://earthexplorer.usgs.gov, accessed on 20 December 2024) captured between March and September 2021. These datasets, originally at 30-m resolution, were standardized to a uniform 1 km scale using a geospatial toolbox to align with other input variables. To minimize redundancy in the statistical framework, variables exhibiting strong pairwise correlations (Pearson’s |r| ≥ 0.8) were excluded during preprocessing. This step led to the removal of numerous bioclimatic variables. Only predictors demonstrating low collinearity were retained for final model development. To predict the geographic ranges of *P. australis* under present and projected scenarios, six bioclimatic parameters (bio1, bio2, bio12, bio14, and bio15) outlined in Table 1 were combined with elevation, LULC, and NDVI datasets. These variables provided the foundation for modeling shifts in the *P. australis*’ habitat over time.

### 4.4. MaxEnt Model

The maximum entropy algorithm (MaxEnt) [46] was chosen due to its reliability for generating precise outputs with limited samples [47]. MaxEnt identifies the most probable distribution of *P. australis* by balancing observed patterns with minimal prior assumptions, ensuring unbiased estimates even when data are sparse. In addition, MaxEnt is particularly advantageous for studies with few observations [48]. For model development, 80% of the *P. australis* location data was randomly allocated for calibration, with the remaining 20% reserved for testing. While default parameters are commonly employed for simplicity, adjustments were made to align with the study’s ecological context, data characteristics, and user expertise to enhance precision [49]. The analysis incorporated 10 independent model runs, each using 500 computational cycles, to generate an averaged probability mode of *P. australis* presence. A total of 550 pseudo-absence points were included to balance the limited occurrence records, with regularization parameters maintained at standard level (i.e., one) [50]. Variables’ relative importance were assessed through a Jackknife procedure, systematically excluding individual data points to test prediction consistency and identify environmental drivers of habitat suitability of *P. australis*. Results were visualized using logistic probability outputs, with thresholds based on minimum training presence values to classify regions into four suitability tiers: unsuitable, low suitable habitat, moderate suitable habitat, and high suitable habitat [51]. These categories were delineated via ArcGIS 10.3.

To evaluate shifts in habitat distribution over time, environmental suitability models were employed to estimate changes. Using geospatial analysis tools within ArcGIS environment, the changes between current and predicted future habitats were quantified. The quantifications were divided into four distinct categories: (i) *Habitat gain*, representing newly viable areas for *P. australis* in subsequent periods; (ii) *unsuitable habitat*, indicating locations unsuitable both currently and in future scenarios; (iii) *no change habitat*, encompassing areas where the species currently thrives and is expected to persist; and (iv) *habitat loss*, identifying regions where the species’ presence is forecasted to diminish.

### 4.5. Model Evaluation

The model’s predictive accuracy was measured using the widely recognized Area Under the Curve (AUC) statistic, a benchmark introduced by Hanley and McNeil [52]. AUC scores range from 0 to 1, with values closer to 1 reflecting strong discriminatory power in distinguishing habitat suitability for *P. australis*. A score of 0.5 indicates random prediction, while values above this threshold demonstrate increasing reliability for differentiating between probable presence and absence zones [30]. Higher AUC values correspond to greater confidence in the model’s ability to classify environmental suitability for *P. australis*.

## 5. Conclusions

The spread of *P. australis* in Iraq mirrors what is happening globally, shifting climates, e.g., annual precipitation, and land-cover features are driving its invasion. As temperatures rise the plant is expanding into new areas under future climate scenarios. Key drivers of *P. australis* prevalence include precipitation, elevation gradients, land-cover and land-use, and vegetation patterns, particularly in wetlands. Targeted interventions should concentrate on the highest-risk zones, which cover less than 1% of the nation’s territory (0.9% or 3994 km^2^), to manage the invasion effectively. This study represents a pioneering assessment of alien invasive plant species geography in Iraq. By mapping current and projected *P. australis* distributions, this research provides baseline data for future surveillance and control programs. Subsequent investigations should incorporate finer-scale climate variables to refine predictive accuracy.

## Figures and Tables

**Table 1:** The model’s input variables, their assigned units and abbreviations, and their quantified impact on *P. australis* habitat predictions are outlined alongside predictive weight (reflecting each factor’s influence on distribution probability).

Variable Used in Modeling	Abbreviation and Unit	Percent Contribution	Permutation Importance
Elevation	DEM (m)	33.2	35.5
wc_bio12	bio12 (annual precipitation) (mm)	31.7	50
landcover2019	LC 2019	19.1	4
wc_bio15	bio15 (precipitation seasonality (coefficient of variation) (mm)	6.1	2.2
wc_bio01	bio1 (annual mean temperature) (°C)	5.1	3.7
wc_bio02	bio2 (mean diurnal range) (°C)	2.1	2.3
IRAQ_NDVI	NDVI	2	2
wc_bio14	bio14 (precipitation of driest month) (mm)	0.7	0.3

**Table 2:** Proportional distributions of suitable and unsuitable habitats for *P. australis* in Iraq under current climatic conditions and projected future scenarios (SSP12.6 and SSP58.5) during two intervals: mid-21st century (2041–2060) and late-21st century (2081–2100) for the MRI-ESM2.0 global model.

	Current Distribution		SSP126_2041–2060 Distribution		SSP585_2041–2060 Distribution	
Class	Area (Km^2^)	Area % (Km^2^)	Area (Km^2^)	Area % (Km^2^)	Area (Km^2^)	Area % (Km^2^)
Unsuitable habitat	366,566.50	85.12	374,098.03	86.87	387,760.80	90.04
Low suitable habitat	51,504.07	11.96	41,818.88	9.71	35,186.10	8.17
Medium suitable habitat	11,540.42	2.68	13,706.59	3.18	6762.17	1.57
High suitable habitat	1021.18	0.24	1008.66	0.23	923.09	0.21
Total area	430,632.17	100	430,632.17	100	430,632.17	100

**Table 3:** Proportional distribution (changes from current to future) of habitat gain and habitat loss for *P. australis* in Iraq under current climatic conditions and projected future scenarios (SSP12.6 and SSP58.5) during two intervals: mid-21st century (2041–2060) and late-21st century (2081–2100) for the MRI-ESM2.0 global model.

	Current to SSP126_2041−2060 Change		Current to SSP585_2041−2060	
Class	Area (Km^2^)	Area % (Km^2^)	Area (Km^2^)	Area % (Km^2^)
Habitat gain	15,403.22	3.58	7823.69	1.82
Unsuitable	351,163.28	81.55	358,742.81	83.31
No change	41,130.91	9.55	35,052.54	8.14
Habitat loss	22,934.75	5.33	29,013.12	6.74
Total area	430,632.17	100	430,632.17	100

## Data Availability

Data are contained within the article.
